# The role of bone marrow derived-mesenchymal stem cells in attenuation of kidney function in rats with diabetic nephropathy

**DOI:** 10.1186/1758-5996-6-34

**Published:** 2014-03-09

**Authors:** Mohamed Talaat Abdel Aziz, Mohamed Abdel Aziz Wassef, Hanan Hosni Ahmed, Laila Rashed, Soheir Mahfouz, Mayssa Ibrahim Aly, Rania Elsayed Hussein, Mai Abdelaziz

**Affiliations:** 1 Unit of Biochemistry and Molecular Biology, Medical Biochemistry Department, Faculty of Medicine, Cairo University, Kasr El Aini, Cairo, Egypt; 2 Pathology Department, Faculty of Medicine, Cairo University, Cairo, Egypt; 3 Internal Medicine Department, Faculty of Medicine, Cairo University, Cairo, Egypt

**Keywords:** Stem cells therapy, Mesenchymal stem cells, Diabetic nephropathy

## Abstract

**Background:**

Stem cell therapy holds a great promise for the repair of injured tissues and organs, including the kidney. We studied the effect of mesenchymal stem cells (MSC) on experimental diabetic nephropathy (DN) in rats and the possible paracrine signals that mediate their action.

**Materials and methods:**

Rats were divided into controls, DN rats, DN rats receiving MSCs. MSCs were given in a dose of (10^6^cells) by intravenous injection. After 4 weeks, 24 h urinary albumin, serum urea and creatinine concentrations, transforming growth factor β (TGF β), tumor necrosis factor α (TNFα), B-cell lymphoma 2 (bcl2) and Bax gene expression and vascular endothelial growth factor (VEGF) were assessed. Histopathology staining was performed.

**Results:**

MSC therapy significantly improved 24 h urinary albumin, serum urea and creatinine concentrations, increased angiogenic growth factor VEGF, and anti-apoptotic protein bcl2 while decreased the pro-inflammatory TNF-α, fibrogenic growth factor TGF β, and pro-apoptotic protein Bax. The histopathology examination showed patchy areas of minimal necrosis and degeneration in renal tubules.

## Background

Diabetic nephropathy (DN) is the most common cause of end-stage renal disease in the world, and could account for disability and high mortality rate in patients with diabetes [1]*.* DN is thought to result from interaction between metabolic and hemodynamic factors. The pathologic changes in DN include renal hypertrophy and extracellular matrix accumulation, which contribute to glomerular sclerosis, which leads to proteinuria and renal failure through the tubular interstitial fibrosis [2].

The basic underlying mechanisms of DN involve high-glucose (HG)-induced production of cytokines and growth factors, which promote leukocyte infiltration, renal cell proliferation, and matrix production [3,4].

Stem cell therapy holds a great promise for the repair of injured tissues and organs, including the kidney. Stem cells are undifferentiated cells that undergo both self-renewal and differentiation into one or more cell types [5].

Among stem cells, mesenchymal stem cells (MSCs) have several advantages for therapeutic use such as ability to migrate to the sites of tissue injury, strong immunosuppressive effects [6,7], and better safety after infusion of allogeneic MSCs [8,9].

Previous studies have shown that MSCs are able to differentiate into several cell types, including cardiomyocytes, vascular endothelial cells, neurons, hepatocytes, epithelial cells, and adipocytes, making them a potentially important source for the treatment of debilitating human diseases. Such multipotent differentiation characteristics coupled to their capacity for self-renewal and capability for the regulation of immune responses, described MSCs as potentially new therapeutic agents for treatment of the complications of diabetes mellitus (DM) [10].

An increasing number of data has showed that the therapeutic effects of MSCs not only rely on their differentiation ability to repair damaged tissue, but also depend on their potency to modulate local environment, activate endogenous progenitor cells, and secrete various factors [11,12].

The present study aims to detect the effect of mesenchymal stem cells on experimental DN in rats and to identify the paracrine signals that mediate MSCs action.

## Materials & methods

### Preparation of the animal model

#### Experimental animals

The study was carried on 60 female albino rats, of an average weight 150–200 gm. Rats were bred and maintained in an air-conditioned animal house with specific pathogen free conditions, and were subjected to a 12:12-h daylight/darkness and allowed unlimited access to chow and water. All the ethical protocols for animal treatment were followed and supervised by the Animal Facilities, Faculty of Medicine, Cairo University. All animal experiments received approval from the Institutional Animal Care Committee.

Animals were divided into 2 groups as follows:

Group 1 (Control group): 20 healthy female albino rats.

Group 2 (Diabetic Nephropathy group): 40 female albino rats in which type1 diabetes was induced by a single intra peritoneal injection of streptozotocin (STZ) [60 mg/kg body weight dissolved immediately before administration in freshly prepared 0.1 mol/L citrate buffer (pH 4.5)]. Diabetes was defined as a random blood glucose reading of >300 mg/dl in 3 continuous days after 72 hours of STZ injection [13]*.* Diabetic nephropathy was confirmed after 12 weeks by measuring serum urea and creatinine in blood and also by histopathological changes.

Group 2 (Diabetic Nephropathy group) was further divided into two subgroups:

Group 2a: consisted of 20 DN rats which received IV PBS.

Group 2b: consisted of 20 DN rats which received MSCs (which were processed and cultured for 14 days), in a single dose of (10^6^cells) per rat by intravenous injection in rat tail vein [14].

Four weeks after MSCs injection, each group was subjected to 24 hours urine collection for urinary albumin concentration measurement, blood sampling through the retro-orbital vein for blood glucose, urea and creatinine concentration estimation.

This was followed by sacrifaction of all groups (by CO_2_ narcosis) to obtain renal tissue specimens. These tissues were examined for:

–Quantitative analysis of TGF β, TNFα, bcl2 and Bax gene expression by real time PCR.

–Histopathological examination of renal tissue by haematoxylin and eosin and by differential stains (massontrichrome MT and periodic acid shift PAS).

–Immunohistochemical examination of VEGF expression.

–Detection of the MSCs homing in kidney tissues after its labeling with PKH26 dye by fluorescent microscope to detect its red fluorescence.

### Preparation of BM -derived mesenchymal stem cells from rats

Bone marrow was harvested by flushing the tibiae and femurs of 6-week-old male white albino rats with Dulbecco’s modified Eagle’s medium (DMEM, GIBCO/BRL) supplemented with 10% fetal bovine serum (GIBCO/BRL). Nucleated cells were isolated with a density gradient [Ficoll/Paque (Pharmacia)] and resuspended in complete culture medium supplemented with 1% penicillin–streptomycin (GIBCO/BRL). Cells were incubated at 37°C in 5% humidified CO2 for 12–14 days as primary culture or upon formation of large colonies. When large colonies developed (80–90% confluence), cultures were washed twice with phosphate buffer saline(PBS) and the cells were trypsinized with 0.25% trypsin in 1 mM EDTA (GIBCO/BRL) for 5 min at 37°C. After centrifugation, cells were resuspended in serum supplemented medium and incubated in 50 cm2 culture flask (Falcon). The resulting cultures were referred to as first-passage cultures [15]. Cells were identified as being MSCs by their morphology, adherence, and their power to differentiate into osteocytes and chondrocytes. Differentiation into osteocytes was achieved by adding 1-1000 nM dexamethasone, 0.25 mM ascorbic acid, and 1-10 mM beta glycerophosphate to the medium. Differentiation of MSCs into osteoblasts was confirmed by morphological changes, Alzarin red staining of differentiated osteoblasts. Differentiation into chondrocyte was achieved by adding 500 ng/mL bone morphogenetic protein-2 (BMP-2;R&D Systems, USA) and 10 ng/ml transforming growth factor b3 (TGFb3) (Peprotech, London) for 3 weeks. In vitro differentiation into chondrocytes was confirmed by morphological changes, Alcian blue staining of differentiated chondrocytes.

### Labeling of MSCs with PKH26

MSCs were labeled with PKH26 supplied by Sigma Company (Saint Louis, Missouri USA). Cells were centrifuged and washed twice in serum free medium. Cells were pelleted and suspended in dye solution. Cells were injected intravenously into rat tail vain. After one month, kidney tissues were examined with a fluorescence microscope to detect and trace the cells.

### Real-time quantitative analyses for TNF-α, TGFβ, bcl2 and Bax gene expression

The relative abundance of mRNA species was assessed using the SYBR Green method using an ABI prism 7500 sequence detector system (Applied Biosystems, Foster City, CA). PCR primers were designed with Gene Runner Software (Hasting Software, Inc., Hasting, NY) from RNA sequences from GenBank (Table 1). All primer sets had a calculated annealing temperature of 60°. Quantitative RT-PCR was performed in duplicate in a 50-μl reaction volume consisting of 2× SYBR Green PCR Master Mix (Applied Biosystems), 2 μl of each primer and 0.5 μl of cDNA. Amplification conditions were 2 min at 50°, 10 min at 95° and 40 cycles of denaturation for 15 s and annealing/extension at 60° for 10 min. The real time- PCR result was analyzed with the step one applied biosystem software. Relative expression of TGF β, TNFα, bcl2 and Bax gene mRNA was calculated using the Livak method. The actual operation of these quantification methods was performed by qPCR software.

**Table 1:** The oligonucleotide primers sequence of studied genes

	**Primer sequence**
TNFα gene	Forward primer: 5′- GACCCTCACACTCAG ATC ATC TTC T -3′
	Reverse primer: 5′- TTGTCTTTGAGATCCATGCCA TT -3′
TGFβ gene	Forward primer: 5′- AATGTCAGCTCAGGAACATCCA -3′
	Reverse primer: 5′- GTTCCTGACACATGAACCCTTG -3′
Bcl2 gene	Forward primer: 5′- GGAGGGCACTTCCTGAG -3′
	Reverse primer: 5′- GCCTGGCATCACGACT -3′
Bax gene	Forward primer: 5′- CTGAGCTGACCTTGGAGC -3′
	Reverse primer: 5′- GACTCCAGCCACAAAGATG -3′
Beta actin	Forward 5′-TGTTGTCCCTGTATGCCTCT-3′
	Reverse 3′-TAATGTCACGCACGATTTCC-5′

### Detection of VEGF by immunohistochemistry

–Unstained positively charged slides were prepared from each paraffin block for immunostaining using monoclonal rabbit anti-human antibody (anti- VEGF, Lab vision, USA. Cat = RB-9072) and ultra-vision detection system (HRP/DAB, Lab vision, USA).

–Positive immunoreactivity to VEGF shows a brown staining in renal endothelial cells of interstitial tissue.

### Biochemical analysis

–Blood was collected from the retro-orbital vein into tubes containing fluoride. Plasma samples were separated by centrifugation at 3000 rpm for 10 min. Plasma glucose was measured by the glucose oxidase method using a commercially available kit (Diamond, Egypt).

–Serum urea and creatinine levels were measured using the conventional colorimetric method using QuantiChrom TM assay kit based on the improved Jung and Jaffe methods, respectively (DIUR- 500 and DICT-500).

–24 h Urinary albumin was assessed by using Albu well M Kit (Murine Microalbuminuria ELISA).

### Analysis of kidney histopathology

Kidney samples were collected in PBS and fixed overnight in 40 g/L paraformaldehyde in PBS at 4°C. Serial 5-μm sections of the cortex and the medulla of the kidney were stained with hematoxylin and eosin (H&E).

### Statistical analysis

Data were expressed as mean ± SD. Significant differences were determined by using ANOVA and post-hoc tests for multiple comparisons using SPSS version 12 computer Software. Results were considered significant at p < 0.05.

## Results

### MSCs culture, identification & homing

**Figure 1:**
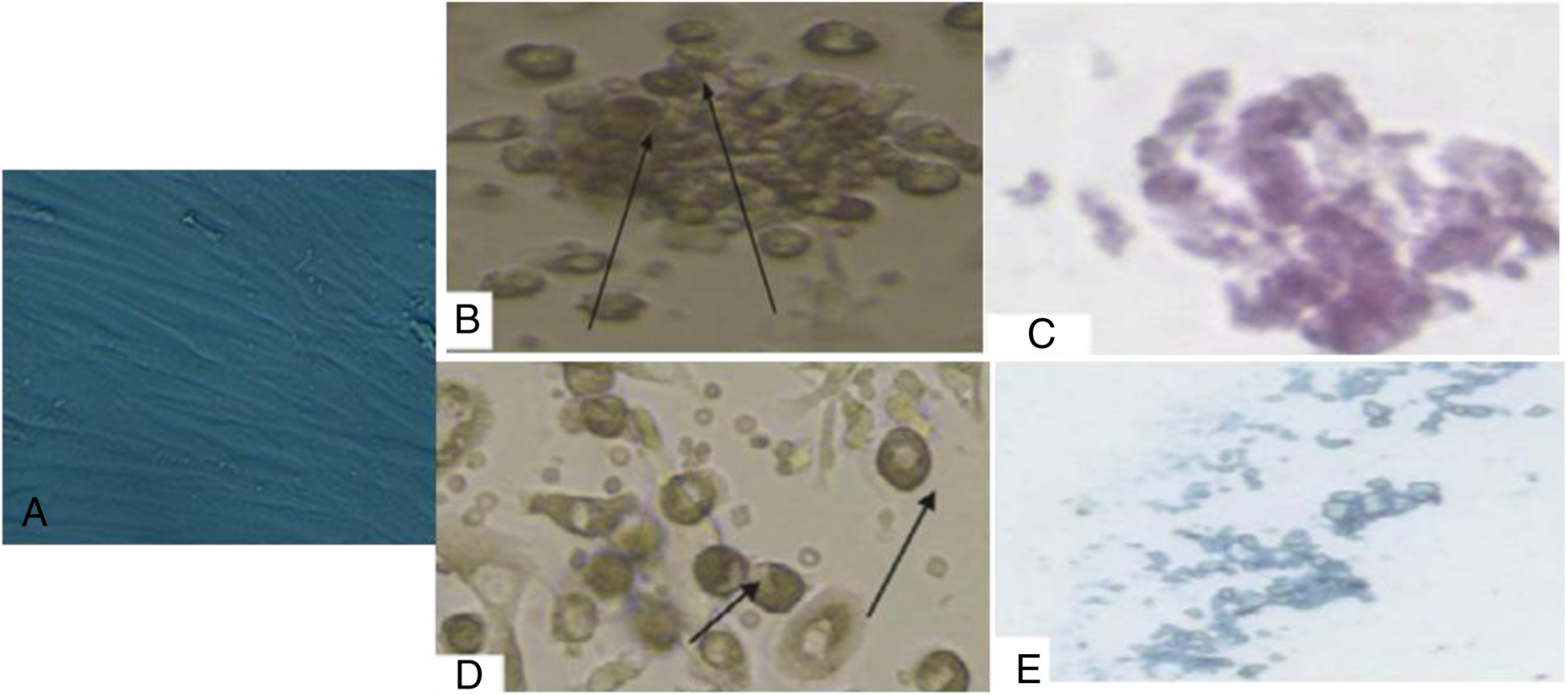
**Morphological and histological staining of differentiated BM-MSCs into osteoblasts and chondrocytes.** undifferentiated MSCs **(A)**, (×20) Arrows for differentiated MSCs osteoblasts after addition of growth factors **(B)**, (×200) MSCs differentiated into osteoblasts stained with Alizarin red stain **(C)**, (×20) Arrows for differentiated MSCs chondrocytes after addition of growth factors **(D)**, (×200) MSCs differentiated into chondrocytes stained with Alcian blue stain **(E)**.

**Figure 2:**
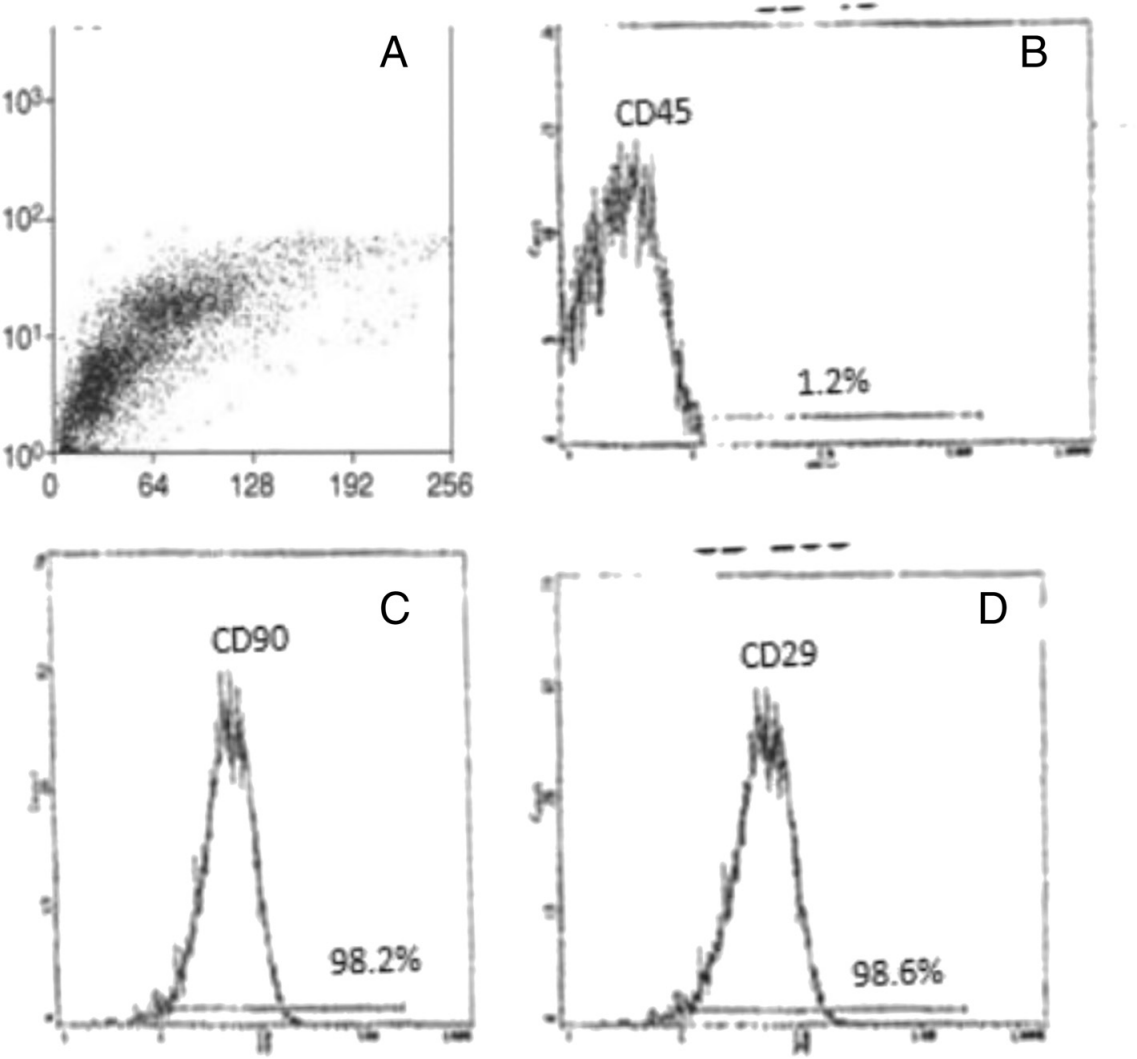
**Characteristics of BM-MSCs.** Cells were stained with the CD45, CD90 &CD29 antibody and analyzed by flow cytometry. BM-MSCs are shown as a dot plot **(A)**. The expression levels of CD45-ve **(B)**, CD90 + ve **(C)** & CD29 + ve **(D)** of BM-MSCs are presented as a histogram. The percentage of expression of the indicated markers was defined in the figure.

**Figure 3:**
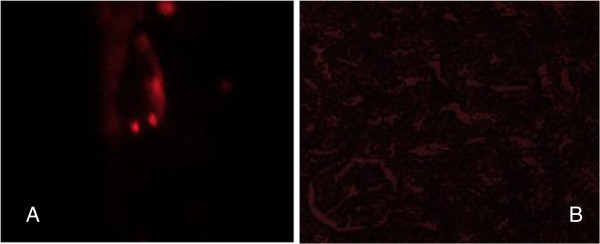
Detection of MSCs labeled with PKH26 fluorescent dye in whole kidney tissue (3A), with phase contrast showed that MSCs distributed in glomeruli and tubules of kidney tissue (3B).

Isolated and cultured undifferentiated MSCs reached 70-80% confluence at 14 days. In vitro osteogenic and chondrogenic differentiation of MSCs were confirmed by morphological changes and special stains (Figure 1A, B,C) and (Figure 1E,F) respectively). In addition, MSCs were identified by surface marker CD45 (−ve), CD90 (+ve ) and CD29 (+ve) detected by flow cytometry (Figure 2B,C and D) respectively. MSCs labeled with PKH26 fluorescent dye were detected in the renal tissues confirming that these cells homed into the kidney tissue (Figure 3A,B).

### MSCs improve the kidney function

The results of the present study show a significant improvement in kidney function. Serum urea and creatinine were decreased in the DN/MSC group compared to the DN group (P = 0.001) as well as 24 h urinary albumin (Table 2).

**Table 2:** Plasma glucose, serum urea, serum creatinine, urinary albumin and body weight in studied groups

**Groups**	**Urea mean ± SD**	**Creatinine mean ± SD**	**Urinary albumin conc.**	**Glucose**	**BW**
	**(mg/dl)**	**(mg/dl)**	**(g/24 h)**	**(mg/dl)**	**(g)**
Control	41.09 ± 2.00	0.20 ± 0.08	0.06 ± 0.02	86.74 ± 6.42	206 ± 6.20
DN	84.11 ± 2.66^#^	0. 96 ± 0.05^#^	28.06 ± 1.92^#^	241.99 ± 43.60#	154 ± 9.60#
DN & MSC	55.79 ± 2.30^#^*	0.55 ± 0.05^#^*	6.11 ± 1.60^#^*	157.86 ± 23.68#*	204.1 ± 4.43*

# Significant p as compared to control group (P < 0.05).

* Significant p as compared to DN group (P < 0.05).

BW = body weight.

### Bcl2 and Bax gene expression

**Figure 4:**
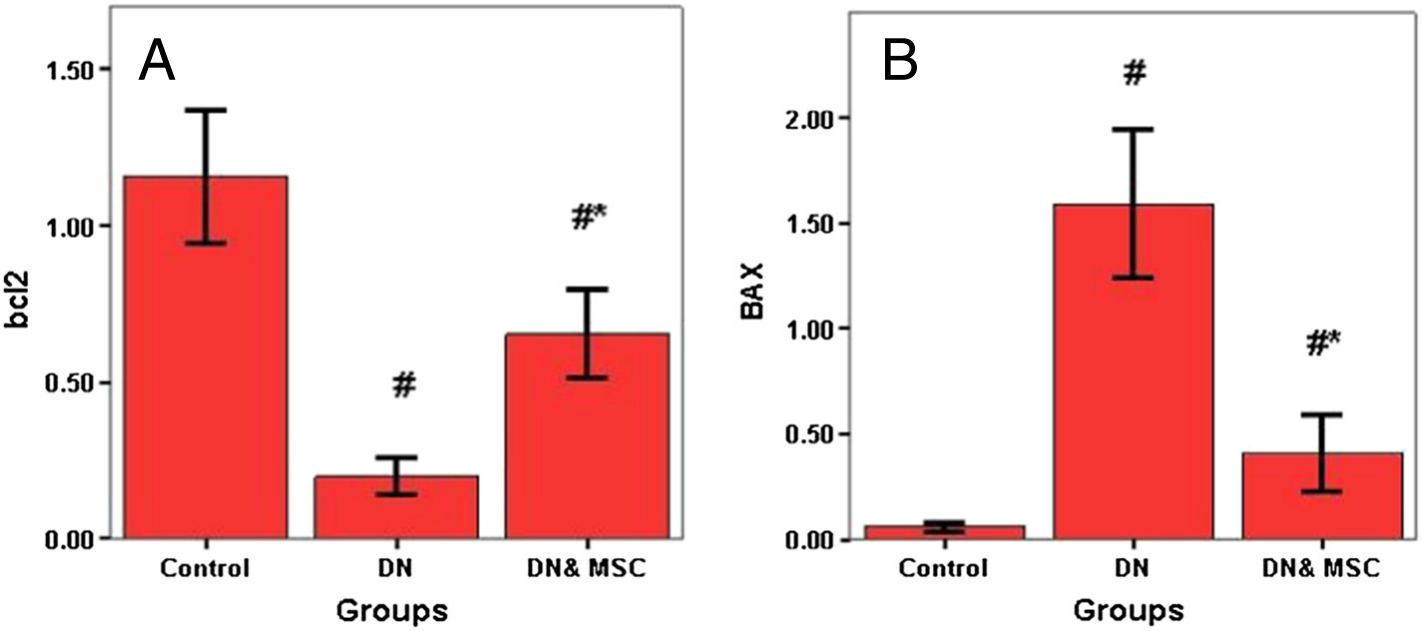
**Quantitave analysis of bcl2 (A), bax (B) gene expression by real time PCR in different groups.** # Significant p as compared to control group (P = 0.001). * Significant p as compared to DN group (P = 0.001).

Bcl2 gene expression was significantly decreased while Bax gene expression was significantly increased in DN group (P = 0.001) compared to control group, whereas Bcl2 gene expression was significantly increase and Bax gene expression was significantly decreased in the DN group that received MSC compared to both control and DN groups (Figure 4). The gene expressions of Bax& Bcl2 were partially reversed following MSCs infusion.

### TGFβ and TNFα gene expression

**Figure 5:**
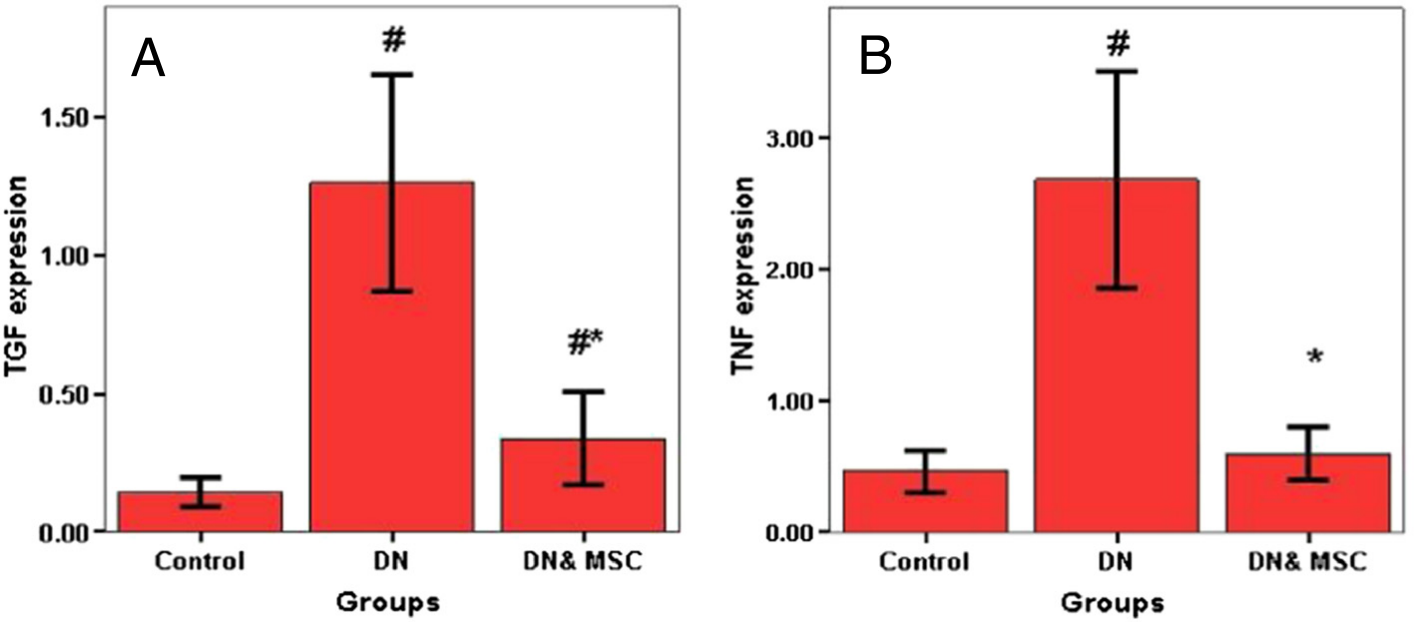
**Quantitave analysis of TGFβ (A), TNFα (B) gene expression by real time PCR in different groups.** # Significant p as compared to control group (P = 0.001). * Significant p as compared to DN group (P = 0.001).

Concerning gene expression, TGFβ and TNFα genes were significantly increased in the DN group (P = 0.001) compared to control group. Whereas their level was significantly decreased in the DN group that received MSC compared to the DN groups (Figure 5). Also, TGFβ and TNFα gene expression showed a positive correlation (P = 0.001 and R value = 0.844) and (P = 0.001and R value = 0.865) with serum creatinine concentration, respectively among the studied groups.

### Histopathological changes

**Figure 6:**
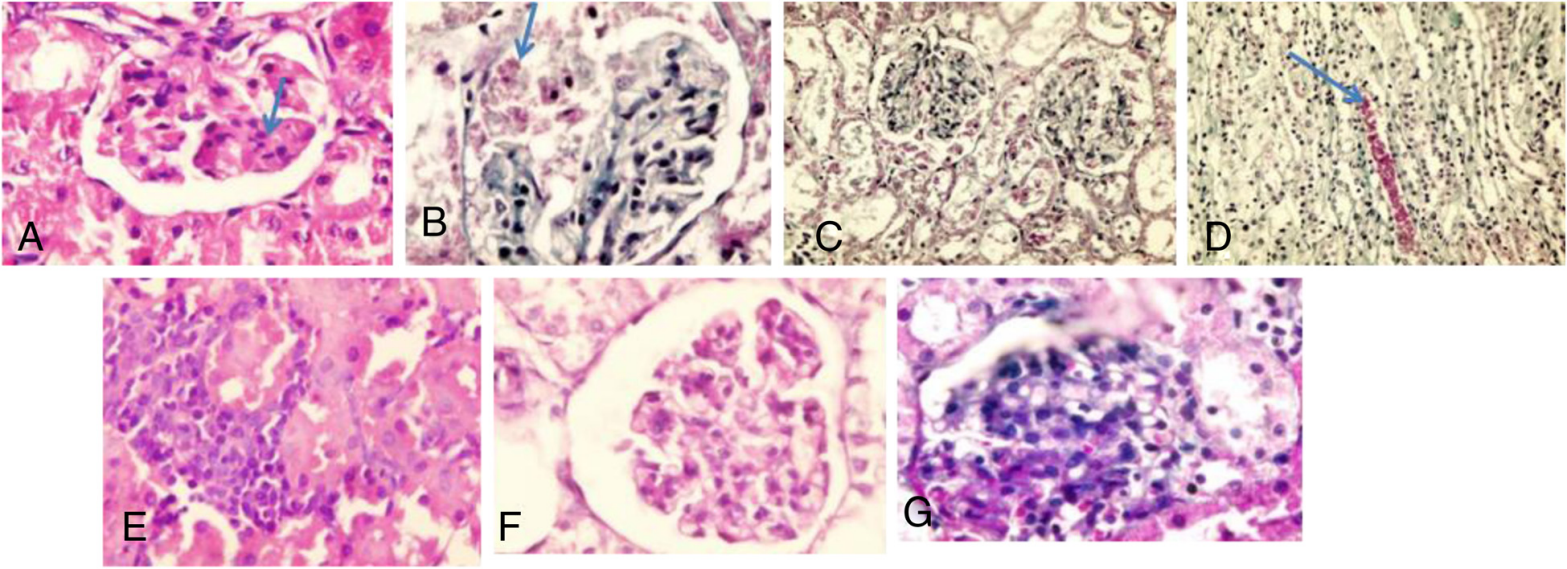
**Histopathological examination of renal tissues in DN group (A, B, C and D) and DN + MSC (E, F and G): (A)** Thickening of glomerular capillary walls &early nodularity (arrow) *(HE ×1000, ***(B)** Fibrin in Bowman’s space (arrow) &green sclerosis of glomerular tuft *(MT ×1000),***(C)** Atrophic changes in cortical tubules with dilatation of lumen & casts *(MT ×400)*, **(D)** Cast in collectingtubule (arrow) *(MT ×400)*, **(E)** Peritubularstem cell collections *(HE ×1000),***(F)** Increased mesangial cells& no thickening of capillary wall *(PAS ×1000),***(G)** Increased cellularity of glomerular tuft & absence of sclerosis *(MT ×1000).*

Histopathological examination of kidney tissues of the DN group showed progressive glomerulosclerosis and tubular damage associated with interstitial fibrosis (Figures 6A, B, C, D). When MSCs were administered, there were small collections of round to oval stem cells insinuating themselves between tubules at the corticomedullary junction (Figure 6E). The glomeruli show decreased congestion of capillary walls and increased mesangial cellularity with diffuse hyaline thickening of glomerular capillary walls (Figure 6F), but in general there were focal milder glomerular changes, absent sclerosis (Figure 6G) and regeneration of tubular epithelium.

### Immunohistochemistry Results

**Figure 7:**
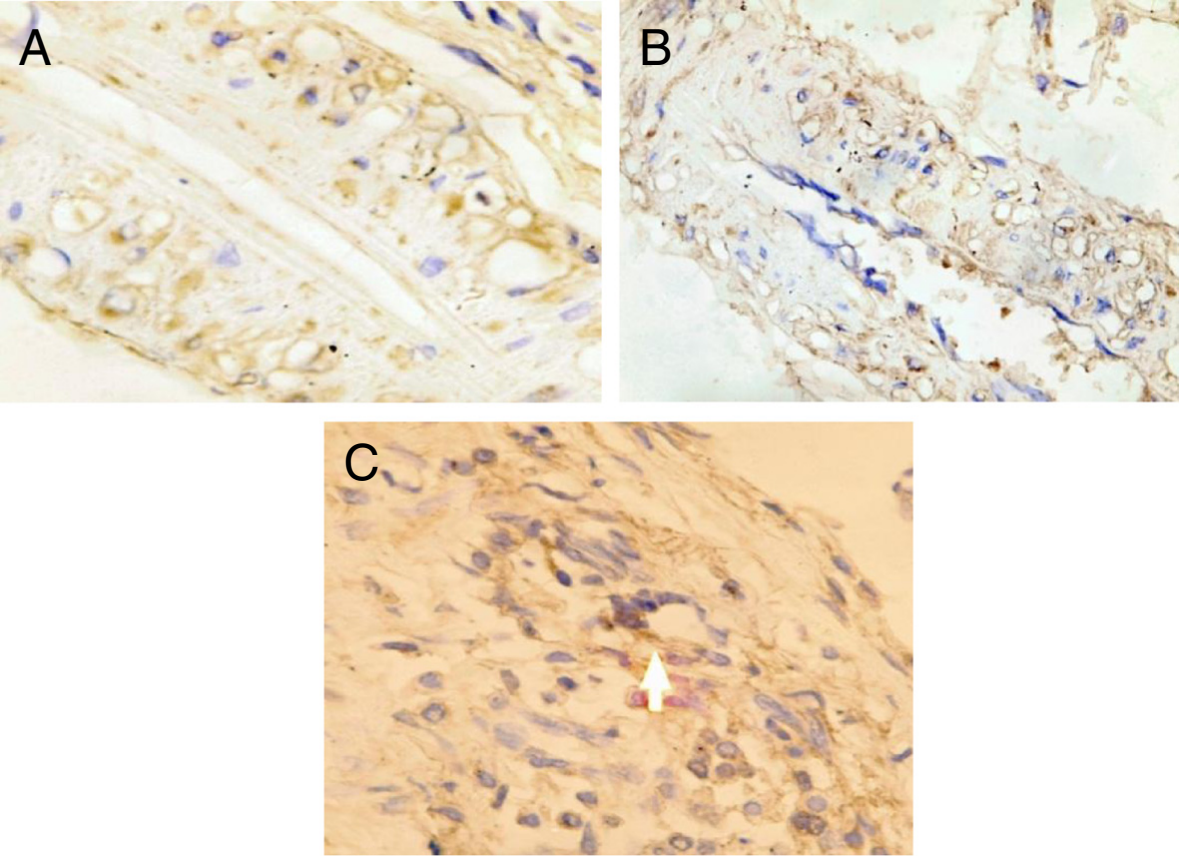
**Immunohistochemistry detection of VEGF: VEGF expression in endothelial cells of interstitial tissue decreased in DN group (7B) compared to the control group (7A).** Following stem cells injection, there was an increase in VEGF expression compared to the diabetic nephropathy group **(Figure**7**C)**.

VEGF was significantly decreased in the endothelial cells of the interstitial tissues in the DN group compared to the control group (7A, B). Following stem cells injection, there was a significant increase in VEGF expression compared to the diabetic nephropathy group (Figure 7C).

## Discussion

Several different growth factors are known to be involved in the development of diabetic complications. Disturbed growth factor signaling adversely affects tissue function and influences the extracellular matrix (ECM). Changes in the amount and composition of ECM are observed in all complications of diabetes and have a central role in their progression [16].

Macrophages are key inflammatory cells mediating kidney inflammation in experimental and human diabetes. Activated macrophages elaborate a host of proinflammatory, profibrotic, and antiangiogenic factors. These macrophage-derived products include but are not limited to TNF-α, IL-1, IL-6, reactive oxygen species (ROS), plasminogen activator inhibitor-1 (PAI-1), matrix metalloproteinases, TGF β, platelet-derived growth factor (PDGF), angiotensin II, and endothelin [17]. In experimental diabetic mice, macrophage accumulation and activation are associated with prolonged hyperglycaemia, glomerular immune complex deposition, increased chemokine production, and progressive fibrosis [18]. In a human study, interstitial macrophage accumulation correlated strongly with serum creatinine, proteinuria, and interstitial fibrosis at the time of biopsy, and inversely with the renal function decline (slope of 1/serum creatinine) over the following 5 years [19]. These human data support animal studies in suggesting a pathological role for macrophages in DN.

TNFα is a potent proinflammatory cytokine and an important mediator of inflammatory tissue damage. TNFα also has an immunoregulatory role [20]*.* Reported actions of TNF-α on renal cells include the activation of second messenger systems, transcription factors, synthesis of cytokines, growth factors, receptors, cell adhesion molecules, enzymes involved in the synthesis of other inflammatory mediators, acute phase proteins, and MHC proteins [21]. This variety of biologic activities results in diverse effects with a significant role in the development of renal damage in diabetes. TNF-α is cytotoxic to renal cells and able to induce direct renal injury [22]. Also TNF-α causes induction of apoptosis and necrotic cell death [23,24], alterations of intraglomerular blood flow and GFR as a result of the hemodynamic imbalance between vasoconstrictive and vasodilatory mediators [25] as well as alterations of endothelial permeability. TNF-α alters the distribution of adhesion receptors involved in cell–cell adhesion (i.e., vascular endothelial-cadherin-catenin complexes) and prevents the formation of F-actin stress fibers. This results in restructuring of the intercellular junction leading to loss of endothelial permeability [26]. On the other hand, TNF-α directly induces reactive oxygen species (ROS) in diverse cells, including mesangial cells [27]. In this study, there was a significant increase in TNFα gene expression in the diabetic nephropathy group compared to the control group. These results were in accordance with Sugimoto et al., [28] who reported a significant rise in the expression of TNF-α in streptozotocin-induced diabetic rat glomeruli after diabetes induction. Advanced glycation end products (AGE), stimulate TNF-α synthesis by renal cells through binding to specific cell surface receptors (RAGE) of the immunoglobulin superfamily identified on several cell types, including renal cells [29,30]. This interaction has been implicated in the development and progression of DN [31], induce a range of biologically important responses, including TNF-α synthesis and secretion [32,33].

TGF-β is a fibrogenic growth factor involved in the pathogenesis of kidney damage and is locally produced in the kidney. It has been shown that TGF-β induces apoptosis of tubular epithelium cells and contributes to progressive renal tubular atrophy [34].

Bone marrow–derived stem cells contribute to cell turnover and repair in various tissue types, including the kidneys [35,36]. MSCs are attractive candidates for renal repair, because nephrons are of mesenchymal origin and because stromal cells are of crucial importance for signaling, leading to differentiation of both nephrons and collecting ducts [37]. In the present study, bone marrow derived mesenchymal stem cells were isolated from male rats, grown and characterized by their adhesiveness and fusiform shape and by detection of CD 29; one of the surface markers of rat mesenchymal stem cells, and were used to detect their possible anti-inflammatory, anti-apoptotic and vascular role in amelioration of renal function in experimental DN model. These cells were actually insinuating themselves into the renal tissue as detected by fluorescent microscope. Similar results have been reported by Morigi et al. [38], who. injected labeled human bone marrow MSCs with PKH 26 dye into mice with induced acute renal failure. The red fluorescence of the MSC was clearly detected in renal tissues.

The possible mechanism by which stem cells improve the kidney function either by fusion or transdifferentiation could not be answered in this study; however, both techniques showed that those cells were able to maintain high population all through the study following MSCs injection. Ling et al., [39] showed 50% replacement of proximal tubular cells with donor cells. Also Rookmaaker et al., [40] declared that bone-marrow-derived cells may home to injured glomerular endothelium, differentiate into endothelial cells, and participate in regeneration of the highly specialized glomerular microvasculature. In addition, they confirmed previous observations that bone-marrow-derived cells can replace injured mesangial cells [41]. Togel et al., [42] stated that infused MSCs were detected in the kidney only early after administration and were predominantly in the glomeruli.

Duffield et al. [43] stated that BMDC contribute in a regenerative cytokine environment in the resulting functional repair. Similarly, bone marrow–derived stem cells seemed to contribute to a relatively small numbers of cells (3 to 22%) to regenerating renal tubular [44] and glomerular cell populations [36]; that is, the majority of reparative cells were derived from intrinsic kidney cells. Regardless the cause, whether it’s MSC differentiation, fusion or merely cytokine induced renal improvement; in this study following MSC injection, there was an improvement of kidney functions, decrease in TNFα, TGFβ, Bax gene expression and increase in bcl2 gene expression and VEGF expression (by immunohistochemistry) in renal tissues. Several studies stated that after 24 h of MSCs infusion, only exceptionally scarce numbers of MSCs were found in the kidney, a pattern that essentially rules out the possibility that significant numbers of infused MSCs are retained in the kidney where they could physically replace lost kidney cells by transdifferentiation. This conclusion is furthermore supported by the fact that there was no intrarenal transdifferentiation events of MSCs within 3 days of administration, whereas occasional MSC-derived capillary endothelial cells were identified only after 5–7 days. From this, it could be deduced that the mechanisms that mediate the protective effects of MSC must be primarily paracrine. This is proved by their expression of several growth and antiapoptotic factors such as VEGF [45,46] and IGF-I, Bax protein [14], all known to improve renal function in CRF, mediated by their antiapoptotic, mitogenic and other cytokine actions. Collectively, these as yet incompletely defined paracrine actions of MSCs result in the renal downregulation of proinflammatory cytokines IL-1β, TNF-α, and IFN-γ [47] and fibrogenic growth factors TGF-β [48] as well as iNOS, and upregulation of anti-inflammatory and organ-protective IL-10 [46,49], as well as bFGF, TGF-α, and Bcl- 2.

Histopathological examination of renal tissue samples of DN & DN after MSCs injection groups supported these results.

In conclusion, MSC are capable of improving the kidney function and regenerating kidney tissues in DN rats most probably through their paracrine action via different growth factors such as VEGF, TGFβ & TNFα and antiapoptotic action via bcl2 & Bax genes.

## Competing interests

The authors declare no competing interest with respect to the authorshipand /or publication of this article.

## Authors’ contributions

MT contributed in study design, manuscript drafting and critical discussion. MA contributed in study design, and critical discussion. HHA contributed in manuscript drafting and critical discussion, LAR contributed in study design, practical work, manuscript drafting and critical discussion. SM contributed in study design, practical work, manuscript drafting and critical discussion. MIA contributed in study design, and critical discussion. REH contributed in manuscript drafting and critical discussion. MA contributed in practical work, manuscript drafting and critical discussion. All authors read and approved the final manuscript.
